# Multidrug Resistance, Biofilm-Forming Ability, and Molecular Characterization of *Vibrio* Species Isolated from Foods in Thailand

**DOI:** 10.3390/antibiotics14030235

**Published:** 2025-02-25

**Authors:** Watcharapong Mitsuwan, Ratchadaporn Boripun, Phirabhat Saengsawang, Sutsiree Intongead, Sumaree Boonplu, Rawiwan Chanpakdee, Yukio Morita, Sumalee Boonmar, Napapat Rojanakun, Natnicha Suksriroj, Chollathip Ruekaewma, Titima Tenitsara

**Affiliations:** 1 Akkhraratchakumari Veterinary College, Walailak University, Nakhon Si Thammarat 80160, Thailand; watcharapong.mi@wu.ac.th (W.M.); ratchadaporn.bo@wu.ac.th (R.B.); sutsiree.in@wu.ac.th (S.I.); sumaree.bo@mail.wu.ac.th (S.B.); rawiwanchan57@gmail.com (R.C.); sumalee.bo@wu.ac.th (S.B.); napapat.ro@mail.wu.ac.th (N.R.); natnicha.sk@mail.wu.ac.th (N.S.); chollathip.ru@mail.wu.ac.th (C.R.); titima.te@mail.wu.ac.th (T.T.); 2 One Health Research Center, Walailak University, Nakhon Si Thammarat 80160, Thailand; 3 Center of Excellence in Innovation of Essential Oil and Bioactive Compounds, Walailak University, Nakhon Si Thammarat 80160, Thailand; 4 Department of Veterinary Medicine, School of Veterinary Medicine, Azabu University, 1-17-71 Fuchinobe, Chuo-ku, Sagamihara 252-5201, Japan; y-morita@azabu-u.ac.jp

**Keywords:** extended-spectrum β-lactamase, multidrug resistance, raw and prepared food, *Vibrio*, whole genome sequencing

## Abstract

Background: *Vibrio* species are common foodborne pathogens that cause gastrointestinal tract inflammation. Multidrug resistance (MDR) in *Vibrio* spp. is a global health concern, especially in aquaculture systems and food chain systems. This study aimed to detect *Vibrio* contamination in food collected from 14 markets in Nakhon Si Thammarat, Thailand, and determine their antibiotic susceptibility. Methods: One hundred and thirty-six food samples were investigated for *Vibrio* contamination. All isolates were tested for antibiogram and biofilm-forming ability. Moreover, the ceftazidime or cefotaxime resistance isolates were additionally investigated for extended-spectrum β-lactamase (ESBL) producers. The isolates were additionally examined for the presence of antibiotic resistance genes. The ESBL-suspected isolates with moderate-to-high biofilm-forming ability were further analyzed for their whole genome. Results: The prevalence of *Vibrio* contamination in food samples was 42.65%, with *V. parahaemolyticus* demonstrating the highest prevalence. Most isolates were resistant to β-lactam antibiotics, followed by aminoglycosides. The overall MDR of isolated *Vibrio* was 18.29%, with an average multiple antibiotic resistance (MAR) index of 16.41%. Most isolates were found to have β-lactam resistance-related genes (*bla*_TEM_) for 41.46%, followed by aminoglycoside resistance genes (*aac*(*6′*)-*Ib*) for 18.29%. Most *Vibrio* showed moderate to strong biofilm-forming ability, particularly in MDR isolates (92.86%). Two ESBL-suspected isolates, one *V. parahaemolyticus* isolate and one *V. navarrensis*, were sequenced. Interestingly, *V. parahaemolyticus* was an ESBL producer that harbored the *bla*_CTX-M-55_ gene located in the mobile genetic element region. While *V. navarrensis* was not ESBL producer, this isolate carried the *bla*_AmpC_ gene in the region of horizontal gene transfer event. Remarkably, the *Inoviridae* sp. DNA integration event was present in two *Vibrio* genomes. Conclusions: These findings impact the understanding of antibiotic-resistant *Vibrio* spp. in food samples, which could be applied for implementing control measures in aquaculture farming and food safety plans.

## 1. Introduction

*Vibrio* species are common foodborne bacteria, and some species are mentioned as pathogenic agents, e.g., *V. cholerae*, *V. parahaemolyticus*, *V. alginolyticus*, *V. vulnificus*, *V. fluvialis*, and *V. furnisii* [1]. The pathogen causes diseases in humans with inflammation of the gastrointestinal tract, wound infections, and septicemia [2]. The main route of *Vibrio* transmission is the consumption of contaminated foods, especially seafood [3]; however, the agents might cross-contaminate from seafood to other foods. Importantly, vibriosis in immunocompromised humans is very important and can be a cause of death [4]. Several countries restricted the importation of foods, especially seafood, for microbial safety, and several foodborne pathogens must not be found, such as pathogenic *Vibrio* spp. [5].

Antibiotic resistance is an important aspect of a global health issue, and antibiotic resistance in *Vibrio* spp. is primarily due to improper use of antibiotics in humans and agricultural fields, particularly aquaculture [6]. Moreover, the improper use of antibiotic drugs in aquaculture systems had a global impact on multidrug resistance (MDR) in *Vibrio* spp. [7,8]. Even if the studies of antibiotic resistance prevalence in *Vibrio* spp. have been served in several areas, the study of their MDR and extended-spectrum β-lactamase (ESBL) production is less. In addition, there are few previous studies about their ESBL phenotypic determination in *Vibrio*, mostly investigating the ESBL-related genes [9,10]. Of this, the phenotypic and genomic characteristics of ESBL-producing *Vibrio* spp. are not well understood and limited. Biofilm is a mechanism of bacteria that adapts themselves to survive in stressful environments [11], such as in antibiotic conditions. Biofilm induces antibiotic resistance by incomplete antibiotic penetration [12]. In addition, several *Vibrio* spp., such as *V. cholerae*, *V. parahaemolyticus*, *V. algninolyticus*, and *V. harveyi*, were found to be proficient biofilm formers [13]. Of this, the formation of biofilm containing antibiotic-resistant *Vibrio* on seafood animals is an important way to transmit antibiotic-resistant strains, and the study of this issue is lacking in southern Thailand.

In Thailand, the outbreak of vibriosis, such as cholera, mainly occurred in areas located near the aquaculture system [14]. Nakhon Si Thammarat is a province in southern Thailand that has numerous aquaculture farms. Interestingly, aquaculture farms use antibiotics at inappropriate dosages to prevent bacterial infection, which has a high chance of inducing antibiotic-resistant bacteria and residue in the animals. Wet markets in Thailand are the places that mainly distribute seafood and meat products to consumers. The characteristic of wet markets is to place the meat, seafood, and vegetables together on the same table or shelf, which may increase the contamination of the foods with pathogens. In addition, the infection of *Vibrio* spp. was continuously reported in Thailand for *V. parahaemolyticus* causing food poisoning and *V. cholerae* causing cholera disease, respectively [15]. Commonly, the presence of ESBL-producing strains was conducted in *Enterobacteriaceae* rather than other bacterial families, which were defined as foodborne pathogens, particularly *Vibrio*. In addition, the study of ESBL–*Vibrio* isolated in Thailand is less, especially in terms of mechanisms of resistance and the association of horizontal gene transfer events and the resistance genes. Of this, ESBL-producing *Vibrio* might be an important source of higher-generation antibiotic drugs, particularly cephalosporins, which are commonly used for the treatment of some infectious diseases in humans.

The objectives of this study were (1) to determine *Vibrio* spp. prevalence isolated from food samples and their cross-contamination of *Vibrio* spp. in non-seafoods collected from markets in Nakhon Si Thammarat province, Thailand; (2) to investigate the occurrence of MDR and ESBL-producing *Vibrio* spp. isolated from foods; and (3) to determine the biofilm-forming ability of *Vibrio* spp. isolated from foods. The findings of the investigations provide useful information for antibiotic drug usage in aquaculture farming and establish the plan for food safety and antibiotic resistance surveillances in southern Thailand. In addition, this study offers the genome characteristics of ESBL-producing *Vibrio* spp. for future research.

## 2. Results

### 2.1. Prevalence of Vibrio Species Isolated from Food Samples

**Figure 1:**
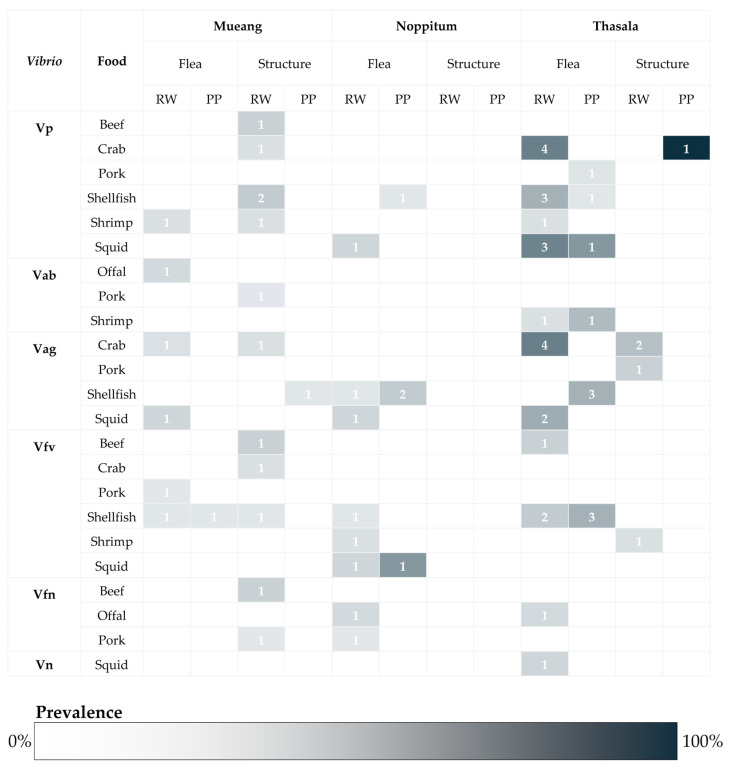
Numbers of samples classified by isolated *Vibrio* species, collected food, food type, district, and type of market; the intensity of color in each block represented the prevalence of discovered species (RW: raw food; PP: prepared food; flea: flea market; structure: structural market; Vp: *V. parahaemolyticus*; Vab: *V. albensis*; Vag; *V. alginolyticus*; Vfv: *V. fluvialis*; Vfn: *V. furnissi*; Vn: *V. navarrensis*).

The overall prevalence of *Vibrio* spp. contamination in 136 food samples was 42.65% (95%CI: 34.21–51.41%), indicating a high level in the Mueang district. Six species of *Vibrio* were identified, including *V. parahaemolyticus*, *V. alginolyticus*, *V. fluvialis*, *V. furnissii*, *V. albensis*, and *V. navarrensis*. The species with the highest prevalence found in examined samples was *V. parahaemolyticus* (16.91%; 95%CI: 11.03–24.29%), followed by *V. alginolyticus* (14.71%; 95%CI: 9.22–21.79%) and *V. fluvialis* (12.50%; 95%CI: 7.45–19.26%). Prevalence of isolated minor species in food samples was 2.94% (95%CI: 0.81–76%) in *V. albensis*, 3.68% (95%CI: 1.20–8.37%) in *V. furnissi* and 0.74% (95%CI: 0.02–4.03%) in *V. navarrensis*. Numbers and prevalences of each *Vibrio* spp. classified by related factors including location of markets, type of food, type of market, and food category are detailed in Figure 1. In this study, the prevalence of *Vibrio* spp. was higher in raw foods (48.31%, 95%CI: 37.59–59.16%) compared to prepared foods (31.91%, 95%CI: 19.09–47.12%). Moreover, the prevalence of *V. parahaemolyticus* was highest (20.22%, 95%CI: 12.45–30.07%) in raw foods, while *V. alginolyticus* (12.77%, 95%CI: 4.83–25.74%) was predominantly found in prepared foods. Interestingly, the prevalence of *Vibrio* contamination in foods bought at structure wet markets (55.17%, 95%CI: 35.69–73.55%) was greater than in flea wet markets (39.25%, 95%CI: 29.95–49.16%). Of this, *Vibrio* contamination in structural markets was more prevalent than in flea markets, with important species including *V. parahaemolyticus*, *V. alginolyticus*, and *V. fluvialis* mostly found. *Vibrio* contamination was primarily found in seafood, particularly crabs (80.00%, 95%CI: 51.91–95.67%), shellfish (56.25%, 95%CI: 37.66–73.64%), squid (64.29%, 95%CI: 35.14–87.24%), and shrimp (36.84%, 95%CI: 16.29–61.64%). Nevertheless, compared to seafood, *Vibrio* contamination is less common in non-seafood products such as beef (33.33%, 95%CI: 7.49–70.07%), pork (18.75%, 95%CI: 7.21–36.44%), and offal (25.00%, 95%CI: 5.49–57.19%). Focused on the seafood group, the prevalence of *Vibrio* contamination in this food group was 55.42% (95%CI: 44.10–66.34%), while that in the non-seafood groups was 22.64% (95%CI: 12.28–36.21%). Of this, seafood, particularly crabs, squids, and shellfish, is the primary source of *V. parahaemolyticus* and *V. alginolyticus* contamination in this study. Moreover, *Vibrio* contamination was primarily found in raw foods (48.31%; 95%CI: 37.59%) rather than in prepared foods (31.91%; 95%CI: 19.09–47.12%), with the three main species being *V. parahaemolyticus*, *V. alginolyticus*, and *V. fluvialis*.

### 2.2. Antibiotic Susceptibility and Antibiotic Resistance Genes of Vibrio Isolates

**Figure 2:**
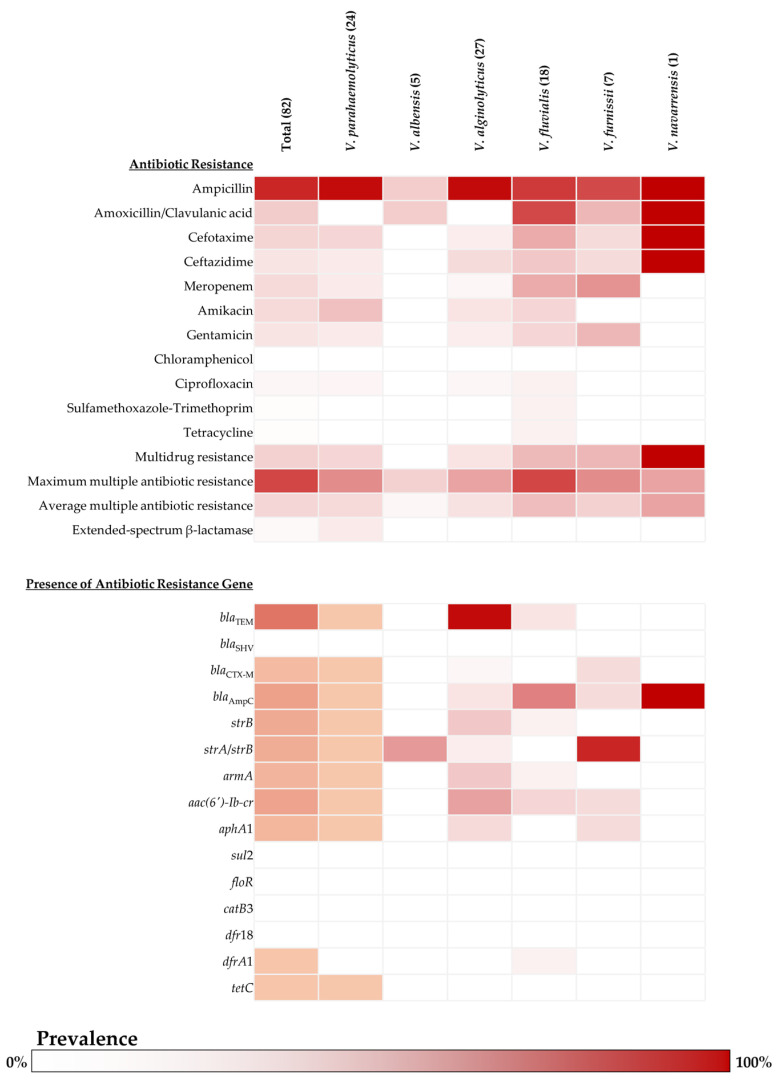
Prevalence and distribution of antibiotic resistance phenotype and carried antibiotic resistance gene of each *Vibrio* species isolated from food (the intensity of color in each block represented the prevalence of isolated *Vibrio* species).

This study found a higher level of resistance to β-lactam antibiotics than other tested classes. Particularly, ampicillin was found to be the most prevalent (85.37%, 95%CI: 75.83–92.20%), and 20.73% of isolated *Vibrio* were resisted to amoxicillin-clavulanic acid (95%CI: 12.57–31.11%). Interestingly, all isolated species showed resistance to ampicillin, which was highly found in *V. alginolyticus* (96.30%; 95%CI: 81.03–99.91%) and *V. parahaemolyticus* (95.83%; 95%CI: 78.88–99.89%). Moreover, one isolated *V. navarrensis* was found to resist most tested β-lactam antibiotics; however, this species had only one isolate for testing. In addition, the isolates were also resistant to other tested β-lactam antibiotics, including cephalosporin (ceftazidime or cefotaxime) and carbapenem (meropenem), which approximately ranged from 10–17%. For the susceptibility of aminoglycosides, approximately 10–14% of isolated *Vibrio* spp. resisted amikacin and gentamicin, which mostly occurred in *V. parahaemolyticus* (25%; 95%CI: 9.77–46.71% for amikacin and 8.33%; 95%CI: 1.03–27% for gentamicin) and *V. fluvialis* (16.67%; 95%CI: 3.58–41.42% of each drug). The MDR of isolated *Vibrio* spp. was 18.29% (95%CI: 10.62–28.37%), with an average MAR index of 16.41% (ranging from 3.64% to 72.73%). Three species showed MDR rates over 15%, including *V. furnissi* (28.57%; 95%CI: 3.67–70.96%), *V. fluvialis* (27.78%; 95%CI: 9.69–53.48%), and *V. parahaemolyticus* (16.67%; 95%CI: 4.74–37.38%). Of this, *V. fluvialis* was found to be a predominant species that revealed the highest value of both the maximum and average MAR index with 72.73% and 26.26%, respectively. The details of antibiotic susceptibility, resistance rate of each tested antibiotic, MDR rate, and MAR index of isolated *Vibrio* spp. are presented in Figure 2. In this study, most isolates were found to carry genes associated with β-lactam resistance, followed by genes related to aminoglycoside resistance. Only two *bla* genes were found in *Vibrio* isolates: *bla*_TEM_ (41.46%; 95%CI: 30.68–52.88%) and *bla*_CTX-M_ (6.10%; 95%CI: 2.01–13.66%). However, no *Vibrio* revealed the presence of *bla*_SHV_; all *bla*_SHV_ suspected amplicons were matched to other proteins in the small multidrug resistance (SMR) family. The SMR gene was detected in 13.41% (95%CI: 6.89–22.74%) of isolated *Vibrio* in this study, which was predominantly found in *V. alginolyticus* (29.63%; 95%CI: 13.75–50.18%), with *V. parahaemolyticus* having a lower prevalence (8.33%; 95%CI: 1.03–27%). Remarkably, *V. alginolyticus* showed the highest prevalence of the *bla*_TEM_ gene (96.30%; 95%CI: 81.03–99.91%) among all the species investigated in this study. In addition, *V. furnissi* revealed the predominant species that harbored the *bla*_CTX-M_ gene (14.29%; 95%CI: 0.36–57.87%). Furthermore, the *bla*_AmpC_ gene was found in 19.51% (95%CI: 11.58–29.74%) of isolated *Vibrio* spp. Notably, *V. fluvialis* is the predominant species related to the presence of the *bla*_AmpC_ gene (50%; 95%CI: 26.02–73.98%). For the prevalence of aminoglycoside resistance-related genes, *strB* gene (14.62%; 95%CI: 7.80–24.17%) and *aac*(*6′*)-*Ib*-*cr* gene (18.29%; 95%CI: 10.62–28.37%) were the most prevalent genes harbored by *Vibrio* spp. isolated in this study. Mainly, the *strB* gene and *aac*(*6′*)-*Ib*-*cr* gene are frequently found in *V. alginolyticus*, *V. furnissi*, and *V. fluvialis*. Determination of aminoglycoside resistance-related genes in *V. parahaemolyticus* revealed a likely low prevalence ranging from 0.04–0.21%, and there were no aminoglycoside resistance-related genes detected in *V. navarrensis*. The prevalence of tetracycline- and trimethoprim-resistant genes was low (1.22%; 95%CI: 0.03–6.61% each), and no isolate harbored a resistance gene for sulfonamide or phenicol. The detection rate of each antibiotic resistance gene determined in this study is presented in Figure 2.

### 2.3. Determination of ESBL Producer and ESBL-Related Genes in Vibrio Isolates

The present study found three *Vibrio* isolates, two of *V. parahaemolyticus* and one of *V. navarrensis*, were suspected of as an ESBL producer through phenotypic testing using third-generation cephalosporins (cefotaxime and ceftazidime) and their combination with a β-lactamase inhibitor (clavulanic acid) (Supplementary Figure S1). Based on the phenotypic confirmatory disc diffusion test (PCDDT) method, all tested isolates were suspected of being ESBL from cefotaxime and its β-lactamase inhibitor; in contrast, no isolates were suspected to be ESBL from ceftazidime and its β-lactamase inhibitor. One ESBL-suspected *V. parahaemolyticus* harbored *bla*_CTX-M_; however, there was no ESBL-suspected *V. parahaemolyticus* in this study that found *bla*_TEM_ and *bla*_SHV_. Therefore, this strain was confirmed as an MDR/ESBL-producing *V. parahaemolyticus* 0610Y harboring the *bla*_CTX-M_ gene. In addition, one ESBL-suspected *V. navarrensis* harbored the *bla*_AmpC_ gene with no ESBL-related gene found, and this isolate finally was not an ESBL producer in this study. Thus, this strain was only defined as MDR-*V. navarrensis* 0706Y harboring the *bla*_AmpC_ gene. Surprisingly, the primers that were mentioned for the detection of *bla*_SHV_, particularly in *Enterobacteriaceae*, were shown to be suited to target the SMR gene region instead of *bla*_SHV_ in all ESBL-suspected *Vibrio* in this study. Furthermore, the PCR products of the *bla*_CTX-M_ gene and the SMR gene were subjected to sequencing for confirmation. The DNA sequences of the *bla*_CTX-M_ gene from ESBL-suspected producers in this study were submitted to the NCBI database.

### 2.4. Biofilm-Forming Ability Determination in Vibrio Isolates

**Figure 3:**
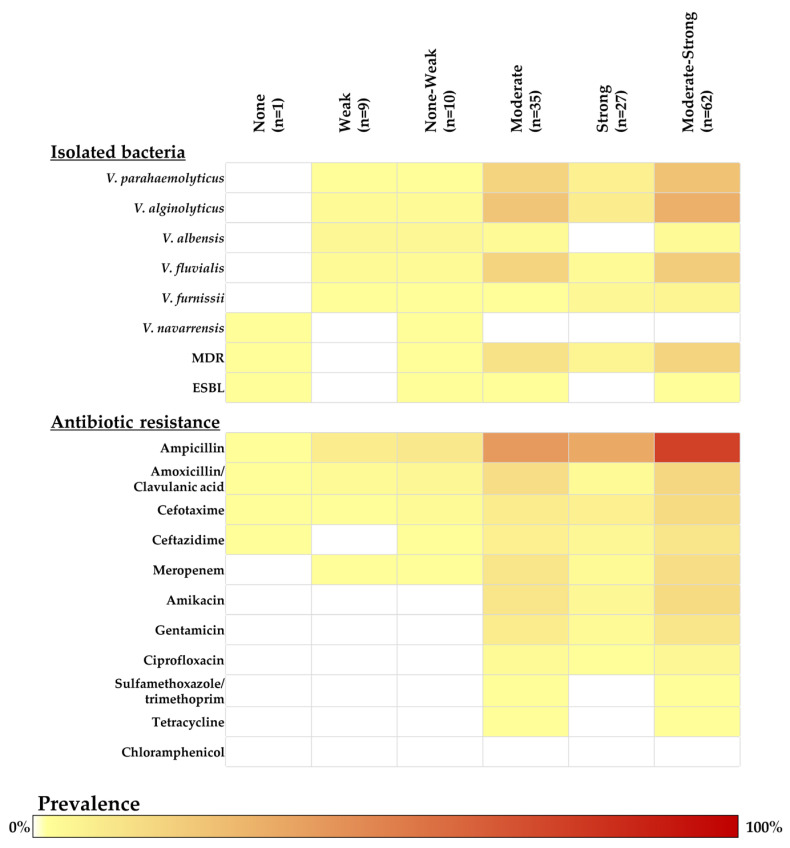
Prevalence and distribution of biofilm forming ability category classified by *Vibrio* species isolated, ESBL type, MDR type, and tested antibiotic drugs. The intensity of color in each block represented the prevalence of categorized biofilm-forming ability. (MDR; multidrug resistance, ESBL; extended spectrum beta-lactamase).

*Vibrio* isolates were tested for their ability to form biofilms using crystal violet assay. Most *Vibrio* isolates in this study showed a moderate ability (48.61%; 95%CI: 36.65–60.69%) to high ability (37.50%; 95%CI: 26.36–49.70%) for biofilm formation. Importantly, most MDR *Vibrio* isolates showed a higher rate of moderate-to-strong biofilm-forming ability (92.86%; 95%CI: 66.13–99.82%). One MDR/ESBL confirmed *V. parahaemolyticus* showed moderate biofilm-forming ability, while one MDR-*V. navarrensis* harboring the *bla*_AmpC_ gene also had moderate biofilm formation. In this study, *V. alginolyticus* (37.10%; 95%CI: 18.20–41.95%) had the most moderate-to-strong ability for biofilm formation compared to other species. In addition, *V. fluvialis* (24.19%; 95%CI: 14.22–36.74%) and *V. parahaemolyticus* (29.03%; 95%CI = 18.20–41.95%) were species that revealed moderate ability for biofilm formation. The details of the biofilm-forming ability of each *Vibrio* are presented in Figure 3.

### 2.5. Antibiotic Resistance Mechanism and Prophage Region Analysis from WGS of MDR/ESBL-Producing Vibrio parahaemolyticus 0610Y and MDR-Vibrio navarrensis 0706Y

**Figure 4:**
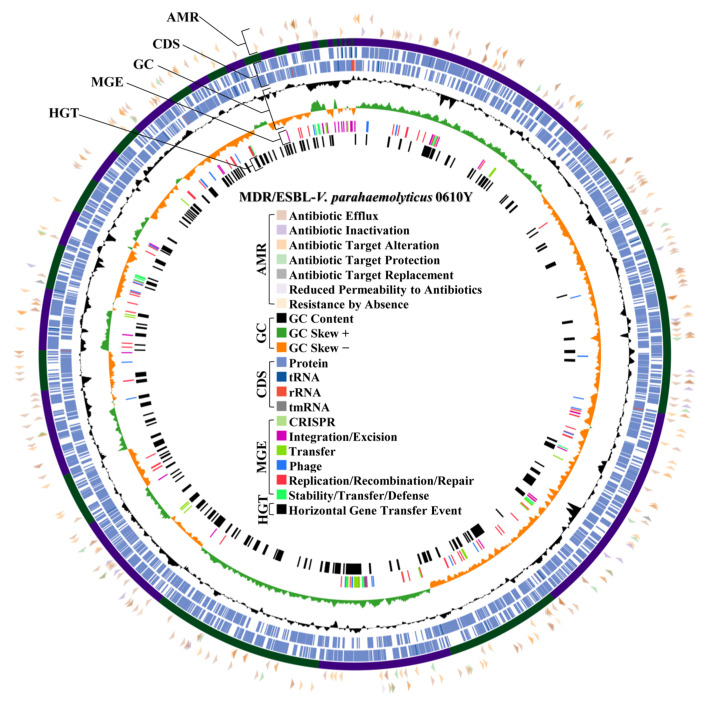
Whole genome and virulence factor regions of MDR/ESBL producing *V. parahaemolyticus* isolate 0610Y with details of antimicrobial resistance gene (AMR) region, coding sequence (CDS) region, GC content and skewness (GC), mobile genetic element (MGE) region, and horizontal gene transfer event (HGT) region.

**Figure 5:**
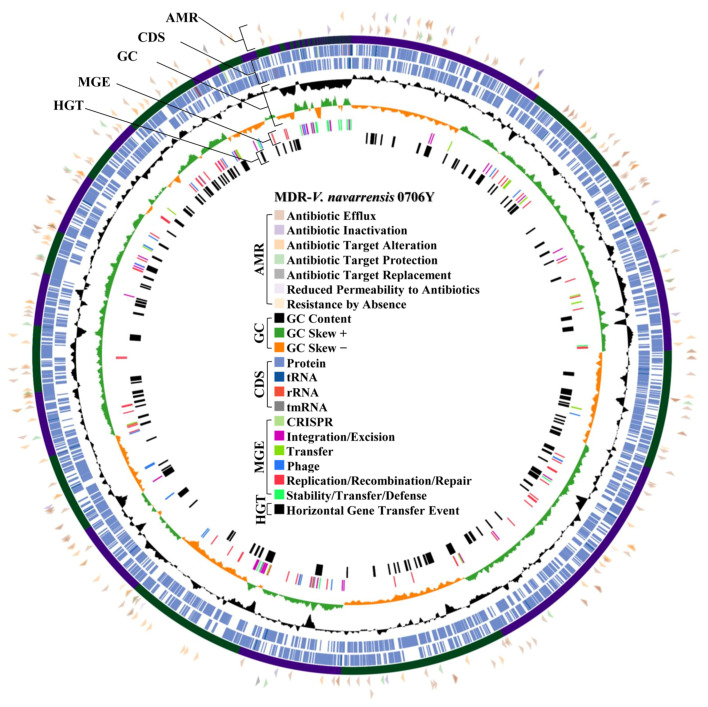
Whole genome and virulence factor regions of MDR-*V. navarrensis* isolate 0706Y with details of antimicrobial resistance gene (AMR) region, coding sequence (CDS) region, GC content and skewness (GC), mobile genetic element (MGE) region, and horizontal gene transfer event (HGT) region.

**Figure 6:**
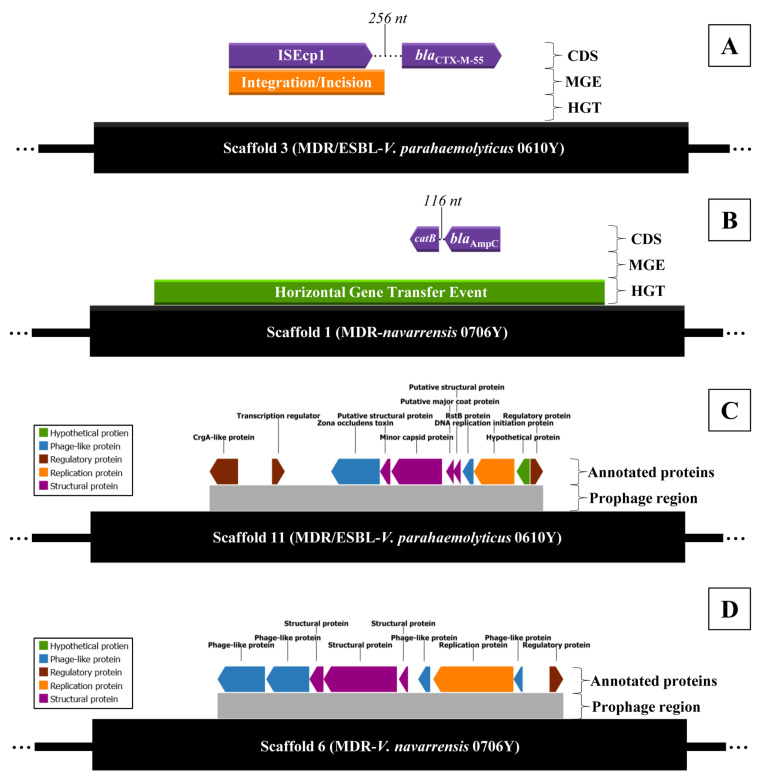
Prediction of mobile genetic element (MGE), horizontal gene transfer event (HGT), and prophage region of MDR/ESBL-producing *V. parahaemolyticus* isolate 0610Y and MDR-*V. navarrensis* isolate 0706Y ((**A**); MGE and HGT of MDR/ESBL producing *V. parahaemolyticus* isolate 0610Y, (**B**); MGE and HGT of MDR-*V. navarrensis* isolate 0706Y, (**C**); prophage region of MDR/ESBL-producing *V. parahaemolyticus* isolate 0610Y, (**D**); prophage region of MDR-*V. navarrensis* isolate 0706Y).

The MDR/ESBL-producing *V. parahaemolyticus* and MDR-*V. navarrensis* with the highest MAR index and moderate-to-strong biofilm formation was selected for WGS. Of this, MDR/ESBL-producing *V. parahaemolyticus* isolate 0610Y (*Vp*0610Y) and MDR-*V. navarrensis* isolate 0706Y (*Vn*0706Y) were included for WGS. The complete genomic architecture of two representative isolates, including GC content, GC skewness, protein coding sequence region, and RNA regions, is mapped in Figure 4 (*Vp*0610Y) and Figure 5 (*Vn*0706Y). Both isolated *Vp*0610Y and *Vn*0706Y were successfully sequenced in their whole draft genome, and the details of the draft genome of each isolated species are presented in Table 1. Based on genomes of sequenced isolates, seven mechanisms of antibiotic resistance gene distribution were revealed in two representative *Vibrio* strains. Of this, 7.05% (95%CI: 6.34–7.81%) and 6.39% (95%CI: 5.67–7.17%) were antimicrobial resistance genes in *Vp*0610Y and *Vn*0706Y, respectively. In brief, both representative *Vibrio* revealed most of the resistance genes related to antibiotic efflux mechanisms for approximately 55% each, followed by antibiotic target alteration around 30% for each. Interestingly, the *bla*_CTX-M-55_ gene was found in the *Vp*0610Y genome, while the *bla*_AmpC_ gene was found in the *Vn*0706Y genome. The distribution of antimicrobial resistance genes classified by the resistance mechanisms is illustrated in Figure 4 (*Vp*0610Y) and Figure 5 (*Vn*0706Y). Moreover, the *bla*_CTX-M-55_ gene found in the *Vp*0610Y genome was found only mobile genetic element in this region. Interestingly, ISEcp1 was found and located near the region of the *bla*_CTX-M-55_ gene of the *Vn*0610Y (Figure 6A). This supported that the *bla*_CTX-M-55_ gene might be transmitted from other bacteria in microbiome. In contrast, the *bla*_AmpC_ gene in the *Vn*0706Y was found to be a horizontal gene transfer event (Figure 6B). The region where the *bla*_AmpC_ gene, located in the *Vn*0706Y genome, was predicted to be a horizontal gene transfer event with other antibiotic resistance genes, including the *catB* gene (Figure 6B). Focused on the region that was annotated as prophage regions, both *Vp*0610Y and *Vn*0706Y were inserted with phage DNA. At the prophage region of both *Vibrio* isolates, there were no antibiotic resistance genes inserting this region. The predicted prophage region was searched in the NCBI virus database, and both genomes revealed *Inoviridae* sp. DNA inserted in the bacterial draft genome. The regions of prophage genes are presented in Figure 6C,D.

## 3. Discussion

*Vibrio* species are one of the most common foodborne pathogens that are found in seafood. The present study found that approximately 33% of the seafood samples were contaminated with *Vibrio* spp., which is a lower percentage compared to a previous study in central Thailand [16]. Mainly, the context of markets between Nakhon Si Thammarat and Bangkok is different, particularly in terms of the freshness of raw seafood between Bangkok and Nakhon Si Thammarat. For Nakhon Si Thammarat, this province is a main source of aquaculture and fishery. Seafood in the market of Nakhon Si Thammarat is sold day to day, and transportation from the source to the market is short, resulting in the restriction of bacterial overgrowth and time for cross-contamination occurrence during transportation being less. Nevertheless, Bangkok is a province in the central region of Thailand, where there is no aquaculture or fisheries. Seafood sold in the markets of this province is imported from other areas. During transportation, the cross-contamination from *Vibrio*-contaminated seafood might occur via cool trucks, ice, or other utensils. This might account for the higher *Vibrio* contamination observed in the previous study performed in Bangkok compared to our study. Interestingly, approximately 30% of prepared foods in this study were contaminated with *Vibrio* spp., possibly due to poor hygiene, water quality, and unclean utensils [17]. Pathogenic *Vibrio* species were isolated from foods in this study and *V. parahaemolyticus* was the predominant pathogenic species found primarily in seafood in this study. Generally, this species was commonly found in marine environments and was identified as a potential agent for gastrointestinal illnesses in humans globally [18], which is mostly related to secretory toxins [17]. In addition, *V. alginolyticus* had the second highest prevalence of pathogenic species in this study, which is a potential human pathogen and is a significant concern for economic and public health. Furthermore, *V. alginolyticus*, mostly found in aquaculture systems [19], can cause disease in patients who contract contaminated water sources for extraintestinal conditions [20]. Notably, *V. alginolyticus* infection, primarily caused by shellfish consumption [21], is a significant concern because of its potential enteritis [22]. *Vibrio fluvialis*, a pathogenic species, was isolated from food samples, especially shellfish, which is considered a public health threa [23] and is a global pathogen causing severe illnesses in humans, including gastroenteritis and extraintestinal infections [24]. The symptoms of *V. fluvialis* are from the effect of enterotoxigenic El Tor-like hemolysin, which is analogous to *V. cholera* [25]. Moreover, *V. albensis*, one of the non-O1 serovars of *V. cholerae* (previously *V. cholerae* biovar *albensis*), was isolated from raw foods in this study. *Vibrio albensis* is a non-O1 serovar *V. cholerae* contaminated in water and sea animals [26], causing severe damage, especially in immunocompromised individuals, despite not often causing clinical disease reports [27]. In this study, *Vibrio furnissii,* not common in non-marine environments, was found in beef, pork, and offal, potentially contaminated by cross-contamination from other seafood products on the same shelf. *Vibrio furnissii* is a non-cholera and pathogenic species that can cause gastroenteritis and extra-intestinal infections in humans [28]. In addition, *V. navarrensis* was found in one raw seafood sample. *Vibrio navarrensis* was reported to be a human pathogenic *Vibrio* by the Centers for Disease Control and Prevention [29].

This study found that most *Vibrio* isolates are resistant to β-lactam classes, especially ampicillin. Particularly, *V. parahaemolyticus* is concerned with the aspect of antibiotic resistance linked to economic and public health threats [30]. Moreover, antibiotic resistance in *V. parahaemolyticus* is a significant concern for humans due to potential gene transfer [31] and excessive antibiotic usage in aquaculture systems [17]. In our study, ampicillin resistance was found as the most common resistance in isolated *Vibrio* for almost all species. Ampicillin was reported as a micropollutant found in water, sludge, and food [32]. Particularly water used in aquatic animal farming, which might be a source of ampicillin micro-pollution in aquaculture system for human food. In addition, ampicillin was also mentioned as an antibiotic used for other food animals other than aquaculture farming [33]. Interestingly, *V. parahaemolyticus* might occur in a high antibiotic resistance rate by the sharing of a resistance genomic island of antibiotic resistance genes from horizontal gene transfer [34]. Moreover, the occurrence of a high antibiotic resistance rate in *Vibrio* might be caused by environmental exposure to the used antibiotics in aquaculture farming. The use of a constant dose of antibiotic drugs directly in water induces the occurrence of antibiotic selective pressure, and the explanation was reported in *V. parahaemolyticus* and *V. vulnificus* [35].

Most β-lactam resistance genes that were found in isolated *Vibrio* were *bla*_TEM_, *bla*_CTX-M_, and *bla*_AmpC_ genes. In this study, MDR *Vibrio* species were found and ESBL isolates were investigated. Interestingly, MDR in *Vibrio* spp., such as *V. cholerae*, *V. fluvialis*, and *V. parahaemolyticus*, is a global concern [23]. The resistance to the β-lactam group, particularly ampicillin resistance, was principally found in MDR isolates in this study, which was similar to other previous studies [36]. *Vibrio species* can obtain antimicrobial-resistant genes from pathogens and intestinal bacteria through mobile genetic elements and horizontal gene transfer, leading to antibiotic resistance [37]. The *bla*_TEM_ gene is classified as a class A β-lactamase, indicating broad substrate hydrolysis potential against penicillins, cephalosporins, and some carbapenems [38]. The CTX-M lactamases, encoded by *bla*_CTX-M_ genes, commonly found in *Enterobacteriaceae*, are found in *Vibrio* spp., particularly *bla*_CTX-M-15_ and *bla*_CTX-M-55_ [39]. The *bla*_CTX-M_ genes are broadly distributed among cephalosporin-resistant *Enterobacteriaceae*, but resistance in foodborne pathogenic *Vibrio* spp. is relatively rare [40]. Furthermore, the *bla*_TEM_ gene is responsible for penicillin resistance, while the *bla*_CTX-M_ gene has the ability to hydrolyze cefotaxime and ceftazidime [41]. For *bla*_AmpC_, this resistance gene is located on chromosomes, while plasmid-associated variations in some bacteria are now widely recognized and distributed through horizontal transfer [42]. In addition, intrinsic *bla*_AmpC_ genes are situated on a mobile genetic element in *Enterobacteriaceae*, which can transmit between diverse bacteria [43]. Interestingly, a report of *bla*_AmpC_ expression in *Enterobacteriaceae* was linked with the hydrolysis of penicillin, amoxicillin, ampicillin, and cephalosporins [44]. Approximately 10% of isolated *Vibrio* spp. were resistant to both amikacin and gentamicin. In addition, most isolates carried *strA*/*strB* and *aac*(*6′*)-*Ib*-*cr* genes, followed by a lesser proportion of *armA* and *aphA1*. The *strA* and *strB* genes are the most prevalent markers of streptomycin resistance, which are encoded to aminoglycoside-3′-phosphotransferase and aminoglycoside-6-phosphotransferase, respectively [45]. Moreover, the *aac*(*6′*)-*Ib* gene, which encodes various forms of an aminoglycoside-acetyltransferase enzyme, has been linked to resistance to amikacin and aminoglycoside modification [46]. Aminoglycoside 3′-phosphotransferase, or APH(3′), is a common enzyme in Gram-negative and Gram-positive organisms, responsible for the phosphorylation reaction of kanamycin [47]. Additionally, the *aphA1* gene, encoding APH(3′)-Ia, was mentioned as an important gene for catalysis of a broad spectrum of antibiotics [48], and the *armA* gene is reported as a responsible aminoglycoside resistance gene against kanamycin, amikacin, and gentamicin [49]. Interestingly, β-lactam and aminoglycoside resistances were found as the most proportion, which were similar to the previous finding in environmental *V. metschnikovii* isolated from leachate ponds [50]. The isolates from the previous study [50] also revealed that environmental *V. metschnikovii* primarily exhibited cefotaxime and ceftazidime resistance, which aligns with our findings. We hypothesized that the resistance to these antibiotic drugs might initially be introduced to environmental strains prior to propagating to other strains. However, the linkage between environmental *Vibrio* spp. and pathogenic strains should be additionally studied.

In this study, more than three-quarters of isolated *Vibrio* spp. presented moderate-to-strong biofilm formation, particularly in MDR isolates. Moreover, greater than 90% of *V. parahaemolyticus* and *V. alginolyticus* had an extremely high proportion of moderate-to-strong biofilm formation. In addition, the isolates that were resisted to β-lactam and aminoglycoside antibiotics had moderate-to-strong biofilm formation in this study. Biofilm formation is an important virulence factor for pathogenic bacteria, such as *V. parahaemolyticus*, which aids in enhancing resistance to antibiotics [51]. In addition, a study mentioned that *V. parahaemolyticus* cells form biofilms to survive, shielding the agent from host defenses and external factors, such as antibiotics, which contribute to antimicrobial resistance [52]. Biofilm cells have greater resistance to antimicrobial agents compared to planktonic cells, therefore enhancing environmental survival, infectivity, and transmission due to their strong biofilm-forming ability [53]. Interestingly, a previous study found that the antimicrobial resistance of *V. parahaemolyticus* biofilm cells to aminoglycoside antibiotics may be linked to increasing biofilm biomass and thickness, and decreasing antibiotic concentrations [54]. Therefore, one of the antibiotic resistance mechanisms of *Vibrio* might be the ability to produce biofilm; however, the deep mechanism should be additionally studied. Interestingly, two isolated species, *V. parahaemolyticus* and *V. navarrensis*, were an MDR/ESBL producer and MDR only, respectively. Most previous studies on ESBL development have focused on the *Enterobacteriaceae* family, with fewer studies performed on the *Vibrionaceae* family. Mostly, ESBL refers to the ability of bacteria to break down penicillin and first-to-third generation cephalosporins; nonetheless, it apparently lacks the ability to particularly disrupt carbapenems [55].

The whole genomes of MDR/ESBL-*V. parahaemolyticus* and MDR-*V. navarrensis* revealed mostly antibiotic resistance genes related to antibiotic efflux pump mechanisms. The energy-dependent efflux pumps of antimicrobial agents in *Vibrio* cells use ATP in primary active transport systems or ion-based electrochemical gradients in secondary active transporters, often called antiporters, which exchange drug and ion during transport [56]. Antibiotics inside *Vibrio* cells were diluted by efflux systems, which actively transported antimicrobial agents to the extracellular environment, allowing bacteria to grow and dominate under high concentrations of antibiotic agents [57]. Notably, *bla*_CTX-M-55_ gene was found in the whole genome of MDR/ESBL-*V. parahaemolyticus*, while *bla*_AmpC_ gene was harbored by MDR-*V. narvarrensis*. Generally, TEM-, SHV-, and CTX-M-type ESBLs are produced by *Enterobacteriaceae* bacteria [58]. In addition, ESBL-related genes (*bla*_TEM_, *bla*_CTX-M_, and *bla*_SHV_) were reported in ESBL-*Vibrio* isolated in Korea and Japan [9,10]. *bla*_CTX-M_ were mentioned as genes related to class A β-lactamase, which is regularly located on plasmids and can easily be transferred to other bacteria by conjugation [59]. Interestingly, a study mentioned that *bla*_CTX-M_ is related to cephalosporin resistance in *V. parahaemolyticus*, where the resistance element can be transferred to other pathogenic organisms [40]. CTX-M-55 is a member of the CTX-M-1 group, which showed stronger hydrolytic activity against ceftazidime [60]. In the *E. coli* study, ESBLs are the primary mechanism of resistance against cephalosporin, with CTX-M-55 producers being known to be resistant to the second and third generations of cephalosporin [61]. Interestingly, the spread of CTX-M-55 genotypes on chromosomes and plasmids might be primarily attributed to mobile elements such as ISEcp1 and IS26 [62], which is similar to this study, which found IS1380 family transpose ISEcp1 closed to the *bla*_CTX-M-55_ region. In a study of *Shigella flexneri*, the ISEcp1 was inserted upstream of the *bla*_CTX-M-55_ gene, supporting the induction of cephalothin resistance [61]. According to other previous studies, the ISEcp1 is responsible for all *bla*_CTX-M_ genotype movement [63] and is a strong activator of *bla*_CTX-M-55_ [62]. Of this, the *bla*_CTX-M-55_ gene found in the genome of *V. parahaemolyticus* in this study might be received from the mobile element transferring event from other bacteria. For the *bla*_AmpC_ gene, the gene located at the horizontal gene transfer region was harbored by MDR-*V. narvarrensis* in this study. The *bla*_AmpC_ gene is chromosomally located, but plasmid-associated variants in some bacteria are becoming more recognized and disseminated through horizontal transfer [42,64]. *Enterobacteriaceae* containing intrinsic *bla*_AmpC_ gene was reported to have the ability to transmit the gene on a mobile genetic element between diverse bacteria [43] and other pathogenic *Vibrio* spp. [65]. Moreover, the expression of the *bla*_AmpC_ gene was mostly stable in penicillins, cephalosporins, and clavulanic acid in *Enterobacteriaceae* [44]. In addition, the *bla*_CMY-2_ gene is mentioned as class C beta-lactamase, similar to *bla*_AmpC_, mostly found in foods and environments. However, there was no blaCMY-2 gene found in the whole genome of MDR-*V. narvarrensis* in this study. Of this, the limitation of this study was the identification of the presence of *bla*_CMY-2_ or other class C beta-lactamase genes other than *bla*_AmpC_.

Finally, a region of prophages was found in each genome of the representative isolates, and the DNA sequence of *Inoviridae* sp. was revealed. The study of this bacteriophage is very limited. Members of the *Inoviridae* family are bacteriophages that have filamentous structures and contain genomes composed of single-stranded DNA. Bacteriophages have the ability to absorb Gram-negative bacteria and integrate their DNA into the bacterial genome [66]. Inoviruses can integrate into the host’s genome through self-encoded integrases or host recombinases, but site-specific integration is not universal [67]. The loss of *Inoviridae* phages is associated with the rapid diversification of bacterial hosts during the spread of epidemics in natural ecosystems, suggesting that widespread inoviruses might play a role in the adaptation of diseases [68]. Shedding of inoviruses may deceive human immune responses and impair the host’s ability to eliminate bacterial infections, leading to increased pathogenicity [69]. The widespread prevalence of inoviruses in *V. parahaemolyticus* and other bacteria, along with limited understanding regarding how the viruses affect the ecology of their hosts, necessitates additional studies [68,70]. Remarkably, these two representative *Vibrio* isolates (MDR/ESBL-producing *V. parahaemolyticus* and MDR-*V. narvarrensis*) might be a potential causative agent because of the evidence of bacteriophage genome integration. A previous study indicated that the shedding of inovirus virions contributes to the biofilm matrix, consequently enhancing virulence and antimicrobial resistance; however, this hypothesis requires further investigation [68]. Moreover, an *in*-*silico* study found that the most common genes encoding β-lactamases, followed by aminoglycoside acetyltransferases, were matched with the most common mechanism of action being antibiotic target replacement, followed by target alteration and target protection [71]. Therefore, the bacteriophage might play an important role in transmitting the antibiotic resistance gene from one isolate to another.

## 4. Materials and Methods

### 4.1. Sample Size and Sampling Techniques

The total sample size of food samples was calculated using a formula that is used to estimate the true prevalence of the event (https://epitools.ausvet.com.au, accessed on 1 October 2024). Furthermore, the prevalence of *Vibrio* spp. in food samples indicates significant variation. Therefore, the estimated prevalence for the purpose of calculating the sample size was set at 50%. In addition, the calculation was conducted using a sensitivity of 95%, a specificity of 90%, a target precision of 10%, and a confidence interval of 90%. A total of 136 food samples were included in this study.

### 4.2. Sample Collection

**Figure 7:**
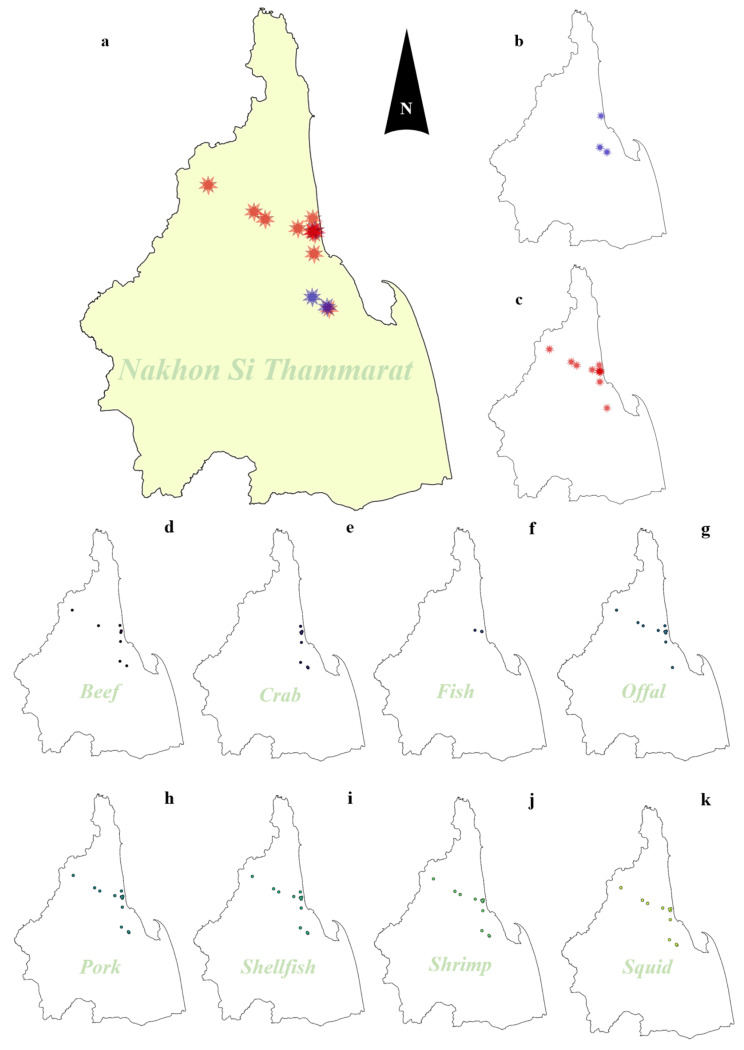
The distribution of wet markets and collected samples in this study ((**a**); all markets in this study, (**b**); structure markets, (**c**); flea markets; (**d**–**k**); distribution of samples in each food type; red star is flea market location; blue star is structural market; colored circle point is market location classified by food sample).

This study covered a total of fourteen wet markets in Mueang district (*n* = 3 markets), Noppitum district (*n* = 3 markets), and Thasala district (*n* = 8 markets). The studied markets were in multicultural areas that were diverse with both Thai and Muslim populations. A total of 136 food samples were obtained from 11 flea markets (*n* = 107 samples) and 3 structured markets (*n* = 29 samples). Furthermore, a variety of food categories were collected, including beef (*n* = 9 samples), crab (*n* = 15 samples), fish (*n* = 3 samples), offal (*n* = 12 samples), pork (*n* = 32 samples), shellfish (*n* = 32 samples), shrimp (*n* = 19 samples), and squid (*n* = 14 samples). Food samples were collected of both raw and prepared foods. Raw foods (*n* = 89) included beef (*n* = 9), crab (*n* = 13), offal (*n* = 11), pork (*n* = 17), shellfish (*n* = 16), shrimp (*n* = 13), and squid (*n* = 10), while cooked foods (*n* = 47) contained crab (*n* = 2), fish (*n* = 3), offal (*n* = 1), pork (*n* = 15), shellfish (*n* = 16), shrimp (*n* = 6), and squid (*n* = 4). The criteria for sample type selection (raw and prepared types) in each market were 8–10 food samples, which included 4–5 raw types and 4–5 prepared types. Nevertheless, in some markets that had some types of food that did not meet the number criteria, another type of food was added instead of increasing the total number of samples to 8–10 per market. The distribution of collected samples in the studied areas is presented in Figure 7.

### 4.3. Vibrio Isolation and Confirmation

Approximately 5 g of food samples were placed into 30 mL of sterile alkaline peptone water (HiMedia^®^, HiMedia Laboratories, Mumbai, India) and then incubated at 37 °C for 18–24 h. The enriched sample was diluted 10-fold and spread on a thiosulfate citrate bile salt sucrose (TCBS) agar (HiMedia^®^, HiMedia Laboratories, Mumbai, India) and then incubated at 37 °C for 18–24 h. The colony that presented a yellow or green appearance on TCBS was re-cultured for further purification. The pure culture of the *Vibrio* suspected colony was re-streaked on trypticase soy agar supplemented with 3% sodium chloride (TSA-NaCl) (HiMedia^®^, HiMedia Laboratories, Mumbai, India). A single colony on TSA-NaCl was picked to confirm and identify a species of *Vibrio* using matrix-assisted laser desorption/ionization time-of-flight mass spectrometry (MALDI-TOF MS) technique of the Bruker Biotyper Microflex LT/SH Maldi-MS system (MALDI Biotyper^®^, Bruker Daltonik GmbH, Bremen, Germany). Briefly, interested colonies were cultivated overnight and then picked into a 1.5-mL microcentrifuge tube for protein extraction following the standard protocol of Bruker Daltonik. The spectrum pattern from MALDI-TOF MS was compared to the pattern in the main spectra library (MSP) library. MALDI-TOF MS for all *Vibrio* suspected colonies was carried out at the Office of Scientific Instruments and Testing, Prince of Songkla University, Thailand.

### 4.4. Antibiotic Susceptibility Testing

The antibiotic susceptibility test of the bacteria was carried out using the disk diffusion method following the instructions of the CLSI M45 edition 3 [72]. Briefly, 3–5 colonies of *Vibrio* spp. were placed into Mueller Hinton Broth (HiMedia^®^, HiMedia Laboratories, Mumbai, India) and incubated at 37 °C for 3–5 h. The sample was adjusted to a turbidity of 0.5 McFarland standard and then spread on Mueller Hinton agar (MHA) (HiMedia^®^, HiMedia Laboratories, Mumbai, India). Eleven antibiotic disks (OxoidTM, Thermo Fisher Scientific, Ely, UK), including ampicillin 10 µg (AMP), amoxicillin 20 µg with clavulanic acid 10 µg (AMC), cefotaxime 30 µg (CTX), ceftazidime 30 µg (CAZ), meropenem 10 µg (MEM), amikacin 30 µg (AK), tetracycline 30 µg (TE), ciprofloxacin 5 µg (CIP), sulfamethoxazole 23.75 µg with trimethoprim 1.25 µg (STX), chloramphenicol 30 µg (C), and gentamicin 10 µg (CN) were placed on the inoculated MHA. The samples were then incubated at 37°C for 16–18 h. The inhibition zone was measured using a vernier caliper, and the interpretation followed the CLSI M45 edition 3 [72]. Following the recommendation of the CLSI M45 edition 3, *Escherichia coli* strain ATCC 25922 was used as the quality control for all susceptibility testing. The interpretation of the antibiotic susceptibility test is divided into three categories: sensitive (S), intermediate (I), and resistant (R).

### 4.5. Determination of ESBL by Phenotypic Confirmatory Disc Diffusion Test (PCDDT)

*Vibrio* isolates that presented cefotaxime and/or ceftazidime resistance were further investigated ESBL phenotype by PCDDT. Briefly, the bacterial culture as described above was spread on MHA, followed by testing with cefotaxime 30 µg/clavulanic acid 10 µg (CEC) and ceftazidime 30 µg/clavulanic acid 10 µg (CAC) (HiMedia^®^, HiMedia Laboratories, Mumbai, India). The samples were then incubated at 37 °C for 16–18 h. The diameter of the inhibition zone of the antibiotic drug and the combination of its β-lactamase inhibitor were measured. Then, the difference in diameter between the antibiotic drug and its β-lactamase inhibitor was subsequently calculated. The difference in inhibition zone between antibiotic drugs and their β-lactamase inhibitors containing antibiotic drugs that presented ≥ 5 mm was defined as an ESBL suspected isolate. In addition, an ESBL-suspected isolate that was presented with at least one ESBL-related gene (*bla*_SHV_, *bla*_TEM_, or *bla*_CTX-M_) was defined as an ESBL-producing isolate. For the quality control for ESBL phenotypic characterization, the previous ESBL-producing *E. coli* received from the other laboratories was used [73].

### 4.6. Biofilm-Forming Ability Determination

The biofilm-forming ability of the isolates was carried out using a crystal violet assay following a previous study [74] with some modification. Briefly, a colony on TSA-NaCl was additionally determined for its biofilm-forming ability using a crystal violet assay. The colonies were cultivated on trypticase soy broth (HiMedia^®^, HiMedia Laboratories, Mumbai, India) with the addition of 3% NaCl and 1% dextrose monohydrate (Loba Chemie™, Mumbai, India) and subsequently incubated at 37 °C for 16–18 h. The cultivated media was adjusted to a concentration of 1 × 10^6^ cells/mL using the supplemented medium. The samples were then transferred to a sterile flat-bottom 96-well plate and incubated at 37 °C for 24 h. The Early Mortality Syndrome (EMS)-causing *V. parahaemolyticus* with strong biofilm-forming ability and bacteria-free medium were used as positive and negative controls, respectively. The media was removed from each well, and then the well was washed twice with 1X phosphate buffered saline (PBS; pH 7.4). The plate was dried at room temperature and then fixed with 96% ethanol for 15 min. The fixative solution was removed, and the well was washed with sterile water. Dried well was stained with 0.1% crystal violet (QRëCTM, Auckland, New Zealand) under a dark condition for 30 min. The excess dye was removed and rinsed with sterile distilled water twice. The plate was dried using air once again prior to the addition of 100% dimethyl sulfoxide and subsequent soaking for 2 h. The plate was measured for optical density at 570 nm (OD570 nm) using a microplate reader (AccuReader M965 Plus, Metertech Inc., Taipei, Taiwan, China). The measurement of the ability for biofilm formation was performed based on a previous study [75], and biofilm-forming ability was classified as none, low, moderate, and strong. Briefly, the biofilm-forming ability of each isolate was compared with the negative control and was included in one category, including (1) OD isolate ≤ OD control for non-biofilm-forming ability; (2) OD control < OD isolate ≤ 2 × OD control; (3) 2 × OD control < OD isolate ≤ 4 × OD control; and (4) 4 × OD control < OD isolate.

### 4.7. Molecular Detection of Antibiotic Resistance Gene

A few colonies of *Vibrio* spp. on TSA-NaCl were picked into sterile distilled water in a sterile tube to extract the genomic DNA using the boiling method. Then, the bacteria-containing solution was boiled for 15 min and then immediately placed on ice for 10–15 min. The tube was centrifuged at 13,000 rpm for 5 min, and 100 µL of the supernatant was separated into a new tube. The extracted DNA was kept at −20 °C until used. Each 25 µL PCR reaction contained MgCl_2_ mixed *Taq* reaction buffer (10X, Excel*Taq*^TM^ series, SMOBIO Technology Inc., Hsinchu, Taiwan, China), *Taq* DNA polymerase (5 U/µL, Excel*Taq*^TM^ series, SMOBIO Technology Inc., Hsinchu, Taiwan, China), dNTPs (0.2 mM each, abm^®^, Applied Biological Material Inc., Richmond, BC, Canada), forward and reverse primers (1 µM each, Macrogen^®^, Seoul, Republic of Korea), and 5 µL of DNA template. The PCR reaction was run using a thermocycler (Eppendorf Vapo Protect, Hamburg, Germany). The details of primer sequences and PCR conditions used to target the antibiotic resistance genes are presented in Supplementary Table S1 [10,63,76,77,78,79,80]. The PCR product was kept at 4 °C until gel electrophoresis. The PCR product was filled with 5 µL of 6X DNA loading dye (ExcelDye^TM^ DNA Loading Dye, SMOBIO Technology Inc., Hsinchu, Taiwan, China). The mixed PCR product was run on a 1.5% agarose gel containing a nucleic acid staining solution (SafeView^TM^ Nucleic Acid Stain, Applied Biological Materials Inc., Richmond, BC, Canada). Each electrophoresis run was conducted under 0.5X tris-acetate-EDTA buffer (120 volts for 30–35 min) with a 100-bp DNA ladder (ExcelBand^TM^ series, SMOBIO Technology Inc., Hsinchu, Taiwan, China). Then, the gel was visualized on a UV illuminator (ChemiDoc^TM^ with Image Lab^TM^ Touch Software Version 2.4, Bio-Rad Laboratories, Hercules, CA, USA). The gel picture was analyzed using Image Lab Standard Edition program version 6.0.1 (Bio-Rad Laboratories, Hercules, CA, USA).

### 4.8. Whole Genome Sequencing (WGS)

ESBL suspected isolates that revealed MDR with the highest multiple antibiotic resistance (MAR) index and moderate-to-strong biofilm formation were selected for whole genome analysis. The genomic DNA of representative isolates was extracted using a commercial kit (BioFACT^TM^ Genomic DNA Prep Kit, BioFACT, Daejeon, Republic of Korea). The quality of the extracted DNA was checked using a microvolume UV-Vis spectrophotometer (ThermoScientific^TM^ NanoDrop^TM^ One Microvolume UV-Vis Spectrophotometer, Madison, WI, USA) and agarose gel electrophoresis. The qualified DNA was then subjected to de Novo sequencing, and the obtained reads were assembled using Velvet version 1.2.10. The contigs were aligned using GapFiller version 1.10, and the assembly of the aligned contigs was performed using SSPACE version 3.0. The scaffolds were used for the prediction of gene-coding regions using Prodigal version 2.6.3. The annotation of gene coding regions was performed using BLAST (https://blast.ncbi.nlm.nih.gov/Blast.cgi, accessed on 1 December 2024), and the E-value < 0.00001 showed significantly best aligned results.

### 4.9. Bioinformatic Analysis

The Comprehensive Antibiotic Resistance Database (CARD) was performed for antibiotic resistance ontology and antimicrobial gene annotation. The annotated genes that were linked to the ESBL phenotype of the bacteria, particularly the β-lactamase gene, found in the genome underwent additional analyses using horizontal gene transfer event prediction and mobile genetic element prediction. Moreover, all whole genomes were predicted for prophage regions using the PHAge Search Tool with Enhanced Sequence Translation (PHASTEST) web server (https://phastest.ca). The Alien Hunter version 1.1.0 and mobileOG-db version 1.1.3 were used as tools for horizontal gene transfer events and mobile genetic element predictions, respectively. The parameters that were set for the Alien Hunter included the ignoring contig that is too short (<20 kbp), motif vectors building (order k) ≤7, and the damping factor = 0.5. In addition, the parameters for the mobileOG-db were set at ≥1 for the number of diamond alignments to report (kvalue), ≥80 for percent of query coverage to sample (queryscore), ≥1 × 10^−5^ for maximum E-score (escore), and ≥60 for percent of identical matches of samples to metadata (pidentvalue). Due to some regions of the bacterial genome that might contain prophage-encoded antibiotic resistance genes [81], the analysis of the prophage region inserted in the whole genome of ESBL-producing *Vibrio* was additionally analyzed. The prophage regions were searched, and genes were confirmed using NCBI virus search (https://www.ncbi.nlm.nih.gov/labs/virus/vssi, accessed on 15 December 2024) and the Basic Local Alignment Search Tool (BLAST) software (https://blast.ncbi.nlm.nih.gov/Blast.cgi, accessed on 15 December 2024), respectively. The parameters that were set for the BLAST include a highly similar sequence optimization program (megablast), 0.05 of the expect threshold, 28 of word size, 1 of match score, and −2 of mismatch score. Finally, the draft genomes were mapped and illustrated using the Proksee software (https://proksee.ca, accessed on 15 December 2024).

### 4.10. Statistical Analysis

All market and laboratory-related factors were recorded on a spreadsheet. The statistical analyses and graphical presentation were performed using R programming language version 4.2.0 (https://www.r-project.org, accessed on 25 December 2024). All statistical analyses were performed under a 95% confidence interval. The testing that presented a *p*-value > 0.05 was considered significant. The resistance isolate number was used to calculate the MAR index following the formula (X/Y), where “X” represents the number of antibiotics to which an isolate was resistant, and “Y” represents the total number of antibiotics tested.

## 5. Conclusions

The present study revealed the prevalence of *Vibrio* spp. isolated from both raw and prepared foods in the southern region of Thailand. Pathogenic species of *Vibrio* were found in the collected food samples with commonly found *V. parahaemolyticus*. The isolated *Vibrio* was greatly resistant to β-lactam and aminoglycoside antibiotics, of which most isolates carried *bla*_TEM_, *bla*_CTX-M_, *bla*_AmpC_, *strA*/*B*, and *aac*(*6′*)-*Ib*-*cr* genes. Interestingly, *V. parahaemolyticus* and *V. navarrensis* were found to be MDR/ESBL-producing isolate and MDR-isolate with moderate biofilm forming ability. In addition, the genome of MDR/ESBL-producing *V. parahaemolyticus* contained the *bla*_CTX-M-55_ gene, while MDR-*V. navarrensis* contained the *bla*_AmpC_ gene. Both draft genomes of representative isolates contained the prophage region of *Inoviridae* sp. Notably, MDR/ESBL-producing *V. parahaemolyticus* and MDR-*V. navarrensis* may potentially be a high-impact pathogen due to the presence of bacteriophage genome integration. The findings of this study contribute to understanding the spread of antibiotic-resistant *Vibrio* spp. carrying antibiotic resistance in food samples and may aid in implementing control measures. The surveillance of antibiotic drug usage in aquaculture farming, food safety plan, and antibiotic resistance surveillance in southern Thailand should be intervened.

## Figures and Tables

**Table 1:** Draft genome characteristics and antimicrobial resistance gene distribution of MDR/ESBL-producing *V. parahaemolyticus* 0610Y (harboring *bla*_CTX-M-55_ gene) and MDR-*V. navarrensis* 0706Y (harboring *bla*_AmpC_ gene).

Feature	MDR/ESBL-*Vp*0610Y	MDR-*Vn*0706Y
1. Draft genome characteristics		
Submitted GenBank assembly	GCA040530925	GCA040530935
Genome size	5.21 Mb	4.62 Mb
GC content	45.5%	48.0%
Number of genes	4794	4224
Protein-coding genes	4620	4030
Protein-coding genes with enzymes	1499	1356
Gene annotation Total antimicrobial resistance genes	338	270
2. Antibiotic resistance mechanism		
Antibiotic efflux	55.65%	55.40%
Antibiotic target alteration	30.14%	30.58%
Antibiotic target protection	6.09%	6.83%
Antibiotic inactivation	4.35%	3.96%
Resistance by absence	1.74%	1.44%
Reduced permeability to antibiotics	1.16%	1.08%
Antibiotic target replacement	0.87%	0.72%

## Data Availability

The original contributions presented in this study are included in the article and Supplementary Materials. Further inquiries can be directed to the corresponding author. The article contains DNA sequence information. The nucleotide sequence data were submitted to GenBank (NCBI) and assigned the following accession numbers: PP559307–PP559310. The draft whole genomes of MDR/ESBL-producing *V. parahaemolyticus* and MDR-*V. navarensis* were assigned as accession numbers: GCA040530925 and GCA040530935, respectively.

## Supplementary Materials

The following supporting information can be downloaded at: www.mdpi.com/article/10.3390/antibiotics14030235/s1; Table S1: Details of the primer sets, and PCR conditions used for molecular detection; Figure S1: Phenotypic characteristics of an ESBL-*Vibrio parahaemolyticus* (A), ESBL-producing *Escherichia coli* (B), and non ESBL producing *E. coli* ATCC 25922 (C) on a Mueller–Hinton agar.
