# Supplementary material for: Multidrug Resistance, Biofilm-Forming Ability, and Molecular Characterization of Vibrio Species Isolated from Foods in Thailand
Source: Antibiotics (Basel). 2025 Feb 25;14(3):235. doi: 10.3390/antibiotics14030235 (PMC11939528; doi:10.3390/antibiotics14030235)
Supplement: Supplementary file 1 [file antibiotics-14-00235-s001.zip › antibiotics-3467874-supplementary.pdf]

Table S1. Details of the primer sets, and PCR conditions used for molecular detection.

Detection	Sequence of primer (5′ → 3′)	Target gene	Annealing temperature	Amplicon size (bp)	Reference
Sulfonamide resistance	Forward: AGGGGGCAGATGTGATCGC<br>Reverse: TGTGCGGATGAAGTCAGCTCC	*sul*2	60.5°C	625	[76]
Florfenicol resistance	Forward: TTATCTCCCTGTCGTTCCAGCG<br>Reverse: CCTATGAGCACACGGGGAGC	*flo*R	60.5°C	526	[77]
Chloramphenicol resistance	Forward: TCAAAGGCAAGCTGCTTTCTGAGC<br>Reverse: TATTAGACGAGCACAGCATGGGCA	*cat*B3	58°C	566	[78]
Trimethoprim resistance	Forward: TGGGTAAGACACTCGTCATGGG<br>Reverse: ACTGCCGTTTTCGATAATGTGG	*dfr*18	60.5°C	389	[76]
Trimethoprim resistance	Forward: CGAAGAATGGAGTTATCGGG<br>Reverse: TGCTGGGGATTTCAGGAAAG	*dfrA*1	60.5°C	372	[77]
Streptomycin resistance gene	Forward: GGCACCCATAAGCGTACGCC<br>Reverse: TGCCGAGCACGGCGACTACC	*str*B	60.5°C	470	[79]
Streptomycin resistance gene	Forward: TATCTGCGATTGGACCCTCTG<br>Reverse: CATTGCTCATCATTTGATCGGCT	*str*A/*str*B	55°C	538	[80]
Aminoglycoside resistance	Forward: TTGCGATGCTCTATGAGTGGCTA<br>Reverse: CTCGAATGCCTGGCGTGTTT	*aac(6′)-Ib-cr*	55°C	482	[80]
Aminoglycoside resistance	Forward: AGGTTGTTTCCATTTCTGAG<br>Reverse: TCTCTTCCATTCCCTTCTCC	*arm*A	53°C	591	[80]
β-lactamase resistance	Forward: CATTTCCGTGTCGCCCTTATTC<br>Reverse: CGTTCATCCATAGTTGCCTGAC	bla_{TEM}	58°C	800	[10]
β -lactamase resistance	Forward: CGCTTTGCGATGTGCAG<br>Reverse: ACCGCGATATCGTTGGT	bla_{CTX-M}	52°C	550	[10]
β -lactamase resistance	Forward: AGCCGCTTGAGCAAATTAAAC<br>Reverse: ATCCCGCAGATAAATCACCAC	bla_{SHV}	58°C	475	[10]
β -lactamase resistance	Forward: TTCTATCAAMACTGGCARCC<br>Reverse: CCYTTTTATGTACCCAYGA	bla_{AmpC}	50°C	550	[63]
Kanamycin resistance	Forward: AAACGTCTTGCTCGAGGC<br>Reverse: CAAACCGTTATTCATTCGTGA	*aph*A1	53°C	500	[80]
Tetracycline resistance	Forward: AACAATGCGCTCATCGT<br>Reverse: GGAGGCAGACAAGGTAT<br>Reverse: CATAACAAACATATGCCCATTTCCG	*tet*C	65°C	1138	[78]

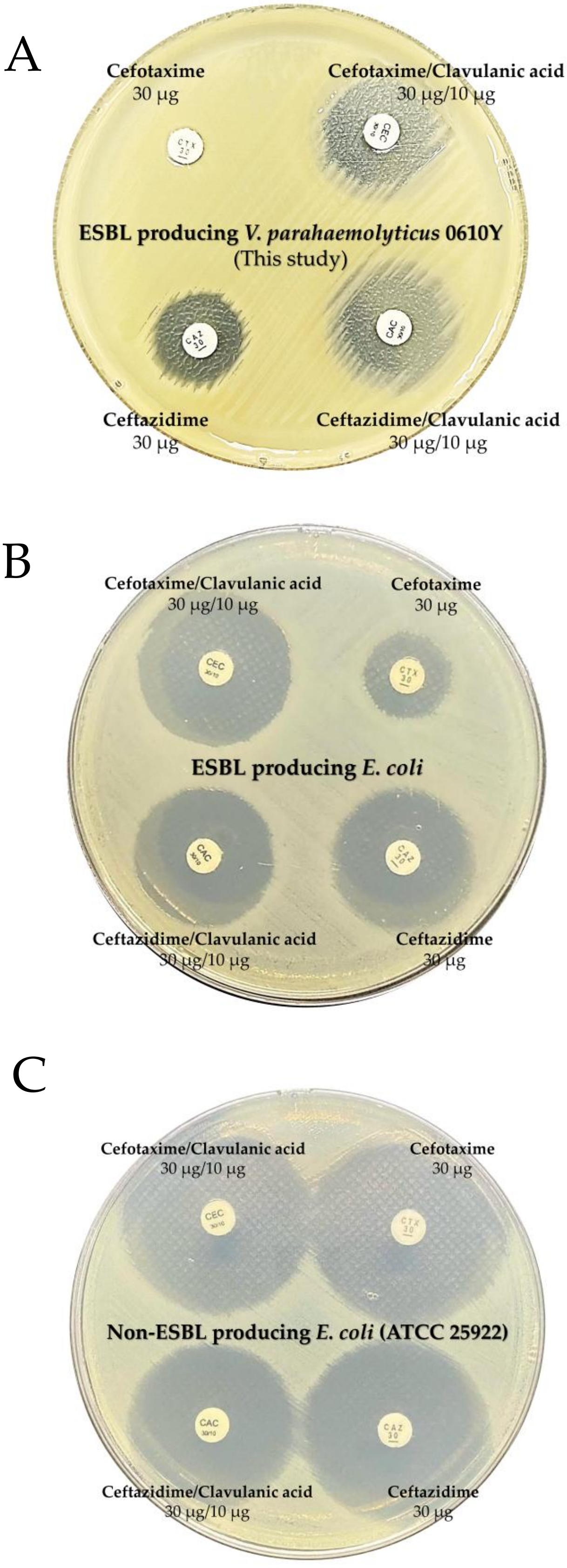

Figure S1. Phenotypic characteristics of an ESBL-*Vibrio parahaemolyticus* (**A**), ESBL-producing *Escherichia coli* (**B**), and non-ESBL producing *E. coli* ATCC 25922 (**C**) on a Mueller-Hinton agar.
